# Time-intensity curve analysis of contrast-enhanced ultrasound is unable to differentiate renal dysfunction in the early post-transplant period - a prospective study

**DOI:** 10.1186/s12882-018-1158-0

**Published:** 2018-12-11

**Authors:** Nordeval Cavalcante Araújo, José Hermógenes Rocco Suassuna

**Affiliations:** 1 University of the State of Rio de Janeiro, Rio de Janeiro, Brasil; 2 Nephrology Section, Hospital Universitário Pedro Ernesto, Boulevard 28 de Setembro, 77 Vila Isabel, 20551-030 Rio de Janeiro, RJ Brasil

**Keywords:** Renal transplantation, Delayed graft function, Contrast enhanced sonography, renal blood flow

## Abstract

**Background:**

Contrast enhanced ultrasonography (CEUS) assessment of kidney allografts mainly focuses on graft rejection. However, studies on delayed graft function (DGF) without acute rejection are still lacking. The aim of this study was to build a time-intensity curve (TIC) using CEUS in non-immunological DGF to understand the utility of CEUS in early transplantation.

**Methods:**

Twenty-eight patients in the short-term postoperative period (<14 days) were divided according to the need for dialysis (early graft function [EGF] and [DGF]) and 37 subjects with longer than 90 days follow-up were divided into creatinine tertiles. Time to peak [TTP] and rising time [RT were compared between groups.

**Results:**

EGF and DGF were similar, except for creatinine. In comparison to the late group, medullary TTP and RT were shorter in the early group as well as the delay regarding contrast arrival in the medulla (in relation to cortex) and reaching the medullary peak (in relation to artery and cortex). In the late group, patients with renal dysfunction showed shorter temporal difference to reach medullary peak in relation to artery and cortex.

**Conclusions:**

Although it was not possible to differentiate EGF and DGF using TIC, differences between early and late groups point to blood shunting in renal dysfunction.

## Background

Since the advent of the contrast enhanced sonography (CEUS) in the early 1980s, many studies have shown the potential of this methodology in different fields of medical imaging [1, 2]. Inspired by successes in imaging the liver, efforts have been made by several researchers to test the utility of this technique in the evaluation of the kidney [3, 4]. In addition to the value of the method for imaging focal lesions [5], great interest has been focused on the assessment of renal blood perfusion [6]. Groups particularly interested in exploring this question have reported encouraging preliminary results in human and animal studies [7–9]. It is therefore not surprising that the utility of CEUS for assessing the perfusion characteristics of kidney allografts has also been investigated [10, 11]. After CEUS examination, the quantification of allograft perfusion by means of time-intensity curve (TIC) analysis enables the measurement of the rate of blood flow in regions of interest (ROI) in different kidney territories [12]. The TIC software allows one to visualize the perfusion curve fitting in a graphical format, enabling parameters based on arrival and peak time of contrast. Accordingly, time to peak (TTP) and rising time (RT) are parameters commonly used to quantify perfusion [13]. The absolute value of a given parameter for a specific kidney region and the difference in its behavior in different kidney regions has been used to characterize allograft dysfunction secondary to acute rejection [12, 14]. However, studies on delayed graft function (DGF) without acute rejection are still lacking.

The aim of this study was to perform CEUS examinations on renal transplant recipients and to interpret the findings on non-immunological DGF (defined as the need for dialysis) in light of current concepts of the pathogenesis of renal ischemia-reperfusion injury. The findings may help us understand the utility of this technique in early renal transplantation.

## Methods

This is a prospective observational study, based on single samples. Two groups of patients were enrolled, one in the short-term postoperative period (less than or equal to 14 days after surgery) comprised of 29 patients who underwent kidney transplantation at our institution in a three-year period from September 2014 to October 2017, and the other comprised of 38 outpatient subjects whose post-transplant follow-up time was greater than or equal to 90 days. All patients from the early group received immunosuppression therapy, consisting of cyclosporine or tacrolimus and mycophenolate mofetil, while in the long-term group, rapamycin, cyclosporine or tacrolimus was given in addition to mycophenolate mofetil or azathioprine. All patients from both groups were given steroids. There were six living donors in the early group and 20 in the late group. All deceased donor kidneys came from heart beating donors.

Some variables associated with the graft were studied. These included the duration of dialysis (time span in months), recipient and donor age and serum creatinine, pre-transplant panel-reactive antibodies, number of human leukocyte antigen (HLA) mismatches, causa mortis and cold ischemia time (CIT). Calculation of the HLA mismatches has been done by the sum of every single mismatch in the A, B and DR loci. The histopathological report of peri-implantation wedge biopsies were also analysed: percentage of glomerular obsolescence (in relation to the number of glomeruli in each biopsy), and presence or absence of interstitial fibrosis and tubular atrophy, interstitial infiltration and edema, vascular lesions (arteriolar hyalinosis, arteriolosclerosis and fibrosis endarteritis), and acute tubular necrosis (ATN).

CEUS examination was performed using a 3.5 MHz convex transducer (Aplio 400; Toshiba; Tokyo, Japan) with a bolus injection of 2.4 ml of Sonovue® (Bracco Int; Milan, Italy) followed by 5 ml of saline solution using a 20-gauge intravenous cannula.

Initially, all patients in our study underwent conventional graft B-mode and Doppler sonography. Assessment of graft size and resistive index (RI) were performed in a sagittal plane with a usual mechanical index (MI) of 1. At least three intrarenal arteries were interrogated to calculate RI and were expressed as means for calculation.

After routine ultrasound, CEUS was performed at a low MI (0.07). The transducer was held in a stable position in coronal plane and the equipment settings (gain, focus position) were kept constant during the procedure. Patients were requested to breath shallow during examination. Data acquisition was documented by digitally storing the images at the start of Sonovue injection over 60s in DICOM format.

**Fig. 1:**
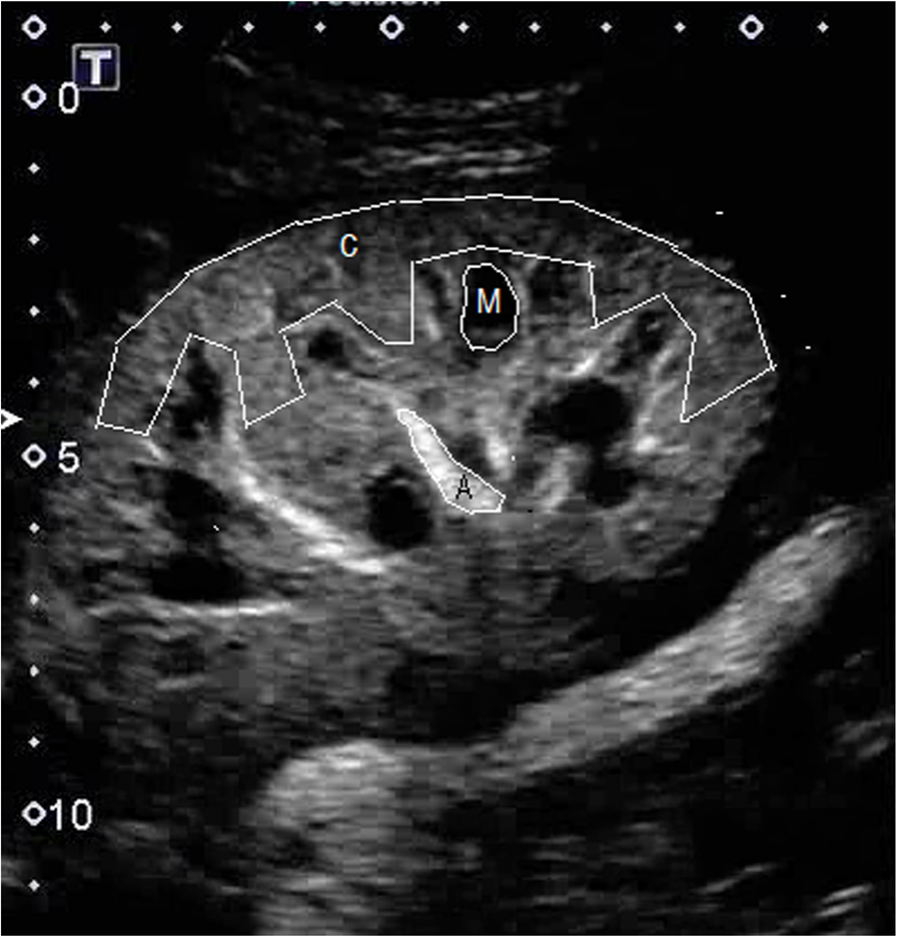
Kidney graft enhancement obtained after administration of contrast media. Spectrum of flow clearly demonstrate diferences in enhancement in three kidney territories. Regions of interest (ROIs) in the kidney were manually outlined by a trackball-guided cursor technique. The anatomical regions are indicated as A, segmental artery; C, cortex; and M, medullary pyramid

**Fig. 2:**
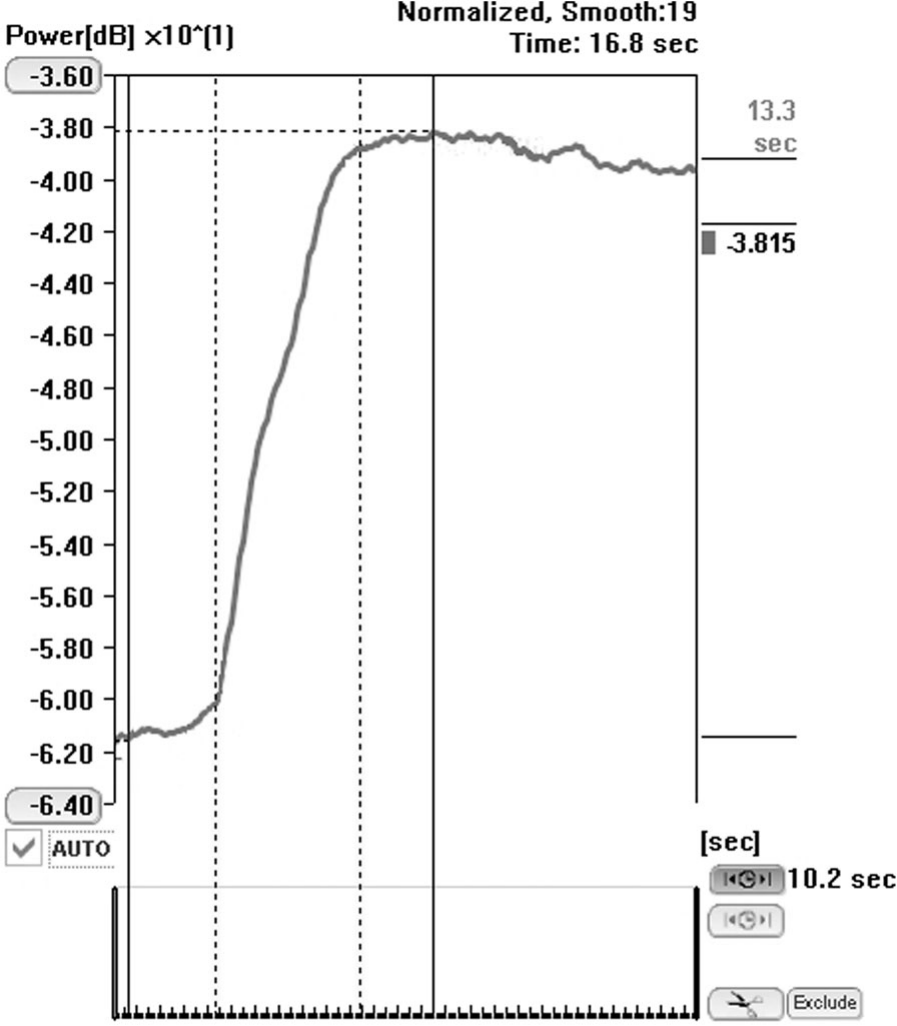
Quantitative analysis using the US system’s inbuilt TIC software. Time to peak (TTP) was calculated according to the corresponding time marks of both nadir (between injection and contrast arrival) and peak enhancement, automatically chosen by the software (solid lines), while rising time (RT) was calculated from the time mark at which a convincing increase in the deflection of the curve was observed until the curve slope clearly flattened (approximately more than 45° relative to a vertical line), chosen by the observer (vertical broken lines)

Regions of interest (ROIs) in the kidney were manually outlined by a trackball-guided cursor technique. ROIs were defined in the area of the segmental artery, medullary pyramid and subcapsular cortex (Fig. 1). Using the freehand “lasso tool” of the software, each ROI was placed over the best visualized artery and medullary pyramid, while the most area of the cortex was selected regardless of the perfusion quality. Since the ROI was adjusted for each territory, the resulting areas were different for each territory in each patient. Since the accuracy of CEUS is limited by tissue motion artifacts, the position of the ROIs was adjusted frame by frame using the tracking tool in the software. Cases in which this procedure was unable to correct motion artifacts were discarded. Subsequently, quantitative analysis with the TIC was used to estimate the time to arrive to and reach the peak of enhancement in three different territories of the graft using the US system’s inbuilt TIC software (Toshiba’s CHI-Q). Time to peak (TTP) was calculated according to the corresponding time marks (vertical) of both nadir (between injection and contrast arrival) and peak enhancement, automatically chosen by the software, while rising time (RT) was calculated from the time mark at which a convincing increase in the deflection of the curve was observed until the curve slope clearly flattened (approximately more than 45° relative to a vertical line), chosen by the observer (Fig. 2). Accordingly, RT represents as much as 80% of the total contrast agent enhancement in the ROI [13]. To calculate TTP, the contrast arrival time of the artery was also used for the cortex and medulla. Using both parameters, it was possible to calculate the temporal difference in contrast arrival (for RT) and peak enhancement time between the artery and the cortex and the medullary pyramid and also between the cortex and the medullary pyramid (for TTP and RT). TTP, RT and delay time between territories were compared between both groups (early and late), in the early group between EGF and DGF patients, and in the late group between stable and renal dysfunction patients and correlated with clinical and histological variables.

### Statistical analysis

Results are presented as mean ± standard deviation for continuous variables and as percentages for dichotomous variables. Groups were compared with the Mann-Whitney test for continuous variables and chi-squared tests for categorical variables. Correlations between continuous variables were assessed by Pearson’s test. Data were analyzed using SPSS software, version 17. Significant differences between groups are indicated by a *p*-value less than 0.05.

The study protocol was approved by the by the institutional ethics committee. All subjects provided written informed consent for participation in the study.

## Results

No adverse effects from the contrast agent were noted. Fourteen out of 81 patients were excluded because of artery stenosis, hydronephrosis, acute rejection and poor study quality (severe breathing motion and cough). The characteristics of the 67 patients eligible for the study are given in Tables 1, 2 and 3.

**Table 1:** Clinical data, laboratory parameters, conventional ultrasound and time-intensity curve parameters of the groups in early transplant period (< 14 days)

	EGF		DGF		
Variable	Mean	SD	Mean	SD	*p* ^a^
Age, y	48.62	13.02	44.13	12.13	0.075
Transplant duration, d	5.85	3.00	7.06	3.40	0.331
Dialysis duration, mos	44.67	42.37	87.25	89.18	0.131
Recipient creatinine, mg/dl	3.47	2.00	6.94	2.23	0.000
Kidney length, mm	120.05	6.77	116.47	9.32	0.245
Main artery RI	0.85	0.08	0.90	0.07	0.086
Intrarenal artery RI	0.74	0.08	0.78	0.10	0.166
PRA class I, %	4.40	13.91	6.07	23.50	0.814
PRA class II, %	3.70	11.70	0.00	0.00	0.221
Donor age, y	44.31	13.68	47.56	16.04	0.692
Donor Creatinine, mg/dl	1.38	0.56	1.84	1.26	0.206
CIT, min	1065	284.08	1185	280.57	0.614
TTP_Arteria	12.33	5.21	12.24	8.47	0.456
TTP_Cortex	16.00	6.09	16.22	7.90	0.759
TTP_Medulla	19.80	5.66	21.54	8.49	0.742
∆t^1^ Peak_AC	3.67	4.51	4.12	3.65	0.469
∆t^1^ Peak_AM	7.47	5.00	9.31	4.12	0.188
∆t^1^ Peak_CM	3.80	2.72	5.19	3.41	0.109
RT_Arteria	6.51	1.80	6.95	3.21	1.000
RT_Cortex	7.75	2.49	8.51	3.96	0.861
RT_Medulla	11.38	2.57	12.30	4.10	0.554
∆t^2^ Arrive_AC	1.40	0.64	1.54	0.59	0.597
∆t^2^ Arrive_AM	2.03	1.19	2.09	0.90	0.597
∆t^2^ Arrive_CM	0.63	0.87	0.56	0.64	0.895
∆t^2^ Peak_AC	2.65	2.83	3.09	2.79	0.303
∆t^2^ Peak_AM	6.91	2.82	7.44	4.28	0.965
∆t^2^ Peak_CM	4.26	4.04	4.35	3.39	0.775

^a^Mann-Whitney test; EGF (early graft function): no need for dialysis; DGF (delayed graft function): need for dialysis, *SD* standard deviation, *RI* resistive index, *PRA* panel reactive antibody, *CIT* cold ischemia time, *TTP* time to peak, ∆t^1^ (temporal difference) calculated based on TTP, *AC* arteria_cortex, *AM* arteria_medulla, *CM* cortex_medulla, *RT* rising time, ∆t^2^: (temporal difference) calculated based on RT

**Table 2:** Clinical data, laboratory parameters, conventional/Doppler ultrasound and time-intensity curve parameters in early (< 14 days) and late (> 90 days) transplant period groups, including the subset of patients from late group with the lowest creatinine tertile

	Early group		Late group		Lowest tertile	
Variable	Mean	SD	Mean	SD	Mean	SD
Age, y	46.14	12.51	46.63	13.74	49.31	11.90
Transplant duration, d	6.52^$&^	3.23	3410	2591	3202	2465
Dialysis duration, mos	69.00	74.90	53.28	59.08	58.83	75.44
Recipient creatinine, mg/dl	5.38^$&^	2.73	2.19	1.12	1.23	0.28
Kidney length, mm	118.08	8.33	117.04	11.93	119.32	6.48
Main artery RI	0.88^$&^	0.08	0.80	0.09	0.77	0.10
Intrarenal artery RI	0.76*^#^	0.09	0.70	0.08	0.68	0.07
PRA class I, %	5.40	19.88	1.29	3.72	2.17	5.31
PRA class II, %	1.48	7.40	8.24	25.13	16.17	39.60
Donor age, y	46.10^&^	14.85	40.91	13.71	31.83	9.38
Donor Creatinine, mg/dl	1.66	1.05	1.80	1.47	1.15	0.87
CIT, min	1138	281.86	1193	186.09	1130	236.04
TTP_Arteria	12.28	7.08	15.09	7.09	15.00	6.45
TTP_Cortex	16.12	7.03	16.09	7.32	15.45	5.99
TTP_Medulla	20.76*^&^	7.29	26.33	9.74	30.35	9.94
∆t^1^ Peak_AC	3.92^$&^	3.99	1.00	2.95	0.45	2.05
∆t^1^ Peak_AM	8.49*^#^	4.55	11.24	8.10	15.35	9.23
∆t^1^ Peak_CM	4.57^$&^	3.14	10.24	7.85	14.89	8.61
RT_Arteria	6.75^$#^	2.64	9.80	4.88	10.07	5.75
RT_Cortex	8.17*	3.34	10.75	5.37	10.44	5.81
RT_Medulla	11.89*^&^	3.47	15.40	6.37	18.85	7.50
∆t^2^ Arrive_AC	1.48	0.61	1.24	1.19	1.05	0.84
∆t^2^ Arrive_AM	2.07*	1.02	2.83	1.33	2.78	1.20
∆t^2^ Arrive_CM	0.59^$&^	0.74	1.59	1.02	1.73	1.08
∆t^2^ Peak_AC	2.89^#^	2.77	2.19	3.17	1.42	2.06
∆t^2^ Peak_AM	7.20	3.64	8.43	6.52	11.57	8.46
∆t^2^ Peak_CM	4.31^#^	3.63	6.24	6.52	10.15	8.32

Mann-Whitney test, *p < 0.05 or ^$^*p* < 0.005 vs. Late; ^#^*p* < 0.05 or ^&^*p* < 0.005 vs. Lowest tertile, *SD* standard deviation, *RI* resistive index, *PRA* panel reactive antibody, *CIT* cold ischemia time, *TTP* time to peak, ∆t^1^ (temporal difference) calculated based on TTP, *AC* arteria_cortex, *AM* arteria_medulla, *CM* cortex_medulla, *RT* rising time, ∆t^2^ (temporal difference) calculated based on RT

**Table 3:** Clinical data, laboratory parameters, conventional ultrasound and time-intensity curve parameters of the lowest and highest creatinine tertiles of late group

	Lowest, Cr < 1.6		Highest, Cr > 2.5		
Variable	Mean	SD	Mean	SD	*p* ^a^
Age, y	49.31	11.90	45.55	13.59	0.642
Transplant duration, d	3202	2465	2200	3003	0.192
Dialysis duration, mos	58.83	75.44	50.64	38.56	0.622
Recipient creatinine, mg/dl	1.23	0.28	3.55	1.10	0.000
Kidney length, mm	119.32	6.48	118.48	15.62	0.870
Main artery RI	0.77	0.10	0.82	0.07	0.400
Intrarenal artery RI	0.68	0.07	0.70	0.07	0.484
PRA class I, %	2.17	5.31	1.00	3.00	0.690
PRA class II, %	16.17	39.60	4.78	14.33	0.690
Donor age, y	31.83	9.38	51.78	6.51	0.000
Donor Creatinine, mg/dl	1.15	0.87	2.05	1.65	0.306
CIT, min	1130	236.04	1272	102.51	0.325
TTP_Arteria	15.00	6.45	14.67	8.83	0.794
TTP_Cortex	15.45	5.99	16.15	8.72	0.977
TTP_Medulla	30.35	9.94	21.56	9.47	0.046
∆t^1^ Peak_AC	0.45	2.05	1.47	2.48	0.486
∆t^1^ Peak_AM	15.35	9.23	6.89	5.01	0.008
∆t^1^ Peak_CM	14.89	8.61	5.42	3.36	0.001
RT_Arteria	10.07	5.75	8.71	3.83	0.794
RT_Cortex	10.44	5.81	10.75	5.73	1.000
RT_Medulla	18.85	7.50	12.81	4.34	0.046
∆t^2^ Arrive_AC	1.05	0.84	1.66	0.79	0.173
∆t^2^ Arrive_AM	2.78	1.20	3.13	1.51	0.685
∆t^2^ Arrive_CM	1.73	1.08	1.46	0.98	0.601
∆t^2^ Peak_AC	1.42	2.06	3.71	4.19	0.045
∆t^2^ Peak_AM	11.57	8.46	7.23	3.76	0.271
∆t^2^ Peak_CM	10.15	8.32	3.52	5.35	0.072

^a^Mann-Whitney test; *SD* standard deviation, *RI* resistive index, *PRA* panel reactive antibody, *CIT* cold ischemia time, *TTP* time to peak, ∆t^1^ (temporal difference) calculated based on TTP, *AC* arteria_cortex, *AM* arteria_medulla, *CM* cortex_medulla, *RT* rising time, ∆t^2^ (temporal difference) calculated based on RT

According to the time after transplantation surgery, the early group comprised 19 patients within 7 days and 10 between 8 and 14 days; in the late group, all 38 patients were more than 90 days post-transplant. Since there were no differences between the two subsets of patients in the early group, except for recipient serum creatinine level (Table 1), they were considered as one group for comparison with the late group.

The late group was further divided in tertiles (reflecting the 33rd and 66th percentiles of distribution) according to the recipient serum creatinine (mg/dl). Thirteen subjects had creatinine < 1.65 (lowest tertile), 12 had creatinine > 1.65 and < 2.4 (middle tertile), and 13 had creatinine > 2.40 (highest tertile). For comparison, the lowest tertile patients were considered to have stable renal function and the highest tertile indicated renal dysfunction.

In the early group, there were no differences between EGF and DGF patients in the analyzed variables, including TIC parameters, except for a lower recipient serum creatinine level in the former group (Table 1). Similarly, there were no differences in the frequency of HLA mismatches, causa mortis, donor type, donor sex, induction agents and maintenance immunosuppression with a calcineurin inhibitor between EGF and DGF (*p* > 0.05). In addition, there were no differences in the frequency of glomerular obsolescence, interstitial fibrosis, interstitial infiltration, acute tubular necrosis and vascular changes in the perioperative biopsy between EGF and DGF (*p* > 0.05).

In comparison to the whole late group, donors were younger, medullary time to peak was shorter, and the temporal differences regarding contrast arrival in the medulla in relation to the cortex and reaching the medullary peak in relation to the artery and the cortex were also shorter in the early group (Table 2). The same comparison using the subset of patients of the late group with creatinine in the lowest tertile yielded even better results from a statistical point of view (Table 2).

When comparing the lowest and highest tertiles, patients with renal dysfunction showed shorter temporal differences in reaching the medullary peak in relation to the artery and the cortex and older age of the donor (Table 3).

## Discussion

Monitoring kidney graft perfusion may theoretically lead to the early recognition of hemodynamic changes and allow for the implementation of adequate preventive and curative strategies that could ultimately limit graft dysfunction or progression. The obligatory surgical and conservation procedures involved in the recovery of deceased donor kidney allografts for transplantation contribute to the occurrence of kidney ischemia-reperfusion injury (IRI) after kidney transplantation [15]. Clinically, IRI in renal transplant manifests as failure of the kidney to function properly, known as DGF when the injury is sufficiently severe. Despite the lack of a consensus definition, DGF is commonly defined as the need for dialysis during the first post-transplant week [16].

Besides being a common cause of DGF, IRI shares many characteristics with post-ischemic acute renal injury in native kidneys [17]. Accordingly, it is reasonable to assume that the two conditions share the same pathophysiological mechanisms. There is a consensus that the initial reduction in RBF triggers the development of an event cascade that ultimately underlies the abrupt and intense reduction in the glomerular filtration rate (GFR) in IRI [18]. However, a short time is necessary for the recovery of RBF [19, 20] or the magnitude of decrement does not parallel GFR reduction in native [21] as well as in allograft kidney [21, 22].

Based on many studies performed in recent decades, there is considerable evidence from experimental animal models as well as humans that a reduction in medullary blood flow contributes to the pathogenesis of acute kidney injury [18, 23, 24]. Therefore, the most important rationale for the assessment of renal blood perfusion and differences in the features of different territories of the graft relies on the known pathophysiological role of RBF changes in acute renal failure secondary to IRI.

The utility of CEUS for the measurement of RBF is comparable with that of other methods. Using TIC parameters derived from intravenous continuous infusion studies, a significant correlation (*r* = 0.69, *P* < 0.005) has been demonstrated between CEUS and RBF determined by PAH clearance in humans [7, 8] and by an ultrasonic flow probe applied directly over the renal artery in dogs [6] and rats [9]. On the other hand, using the bolus technique, contrast enhanced sonography has provided renal perfusion results similar to those obtained with technetium Tc 99 m diethylenetriamine pentaacetic acid in human renal transplant recipients [25] and with laser-Doppler flowmetry in mice [26].

The current study examined how the use of CEUS may help non-invasively differentiate EGF and DGF in renal graft recipients early after implantation by establishing the linkage between the need for dialysis and the results of TIC analysis. The work yielded two main results. First, it was not possible to differentiate EGF and DGF (excluding acute rejection) patients using TTP or RT derived from TIC analysis performed in three kidney territories (segmental artery, cortex and medulla). This is in accordance with a previous report that found no perfusion quotient difference between non-dialyzed patients (slow graft function) and DGF patients with biopsy-proven acute tubular necrosis [27]. The similarities in the TIC analysis of EGF and DGF patients were further supported by the absence of differences in TTP and RT between early acute tubular necrosis (0.21 months) and stable patients (8.31 months) [13]. Conversely, using the bolus technique in the early posttransplant period, acute renal transplant rejection patients in comparison with non-rejecting patients showed an increase in cortex TTP [14, 28], a delay in the first conspicuous increase in the value between the renal cortex and the main renal artery [10], and a delay in the maximum value of the inflow from the segmental arteries to the cortex and to the pyramids [12]. An increase in medullary RT and TTP has also been reported in late (> 20 months) acute rejection compared to stable patients (8 months) [13].

The findings of the present study are consistent with those that found similar results using other methods. Indeed, no differences in RBF have been found between immediate graft function (or recovering acute renal failure) and delayed graft function (or sustained acute renal failure) in allograft recipients, using Doppler flowmetry [29] or *p*-aminohippurate and phase contrast cine-magnetic resonance imaging methods [17, 22].

It is reasonable to keep in mind that the two groups (EGF and DGF) have been exposed to the same conditions (pre-donation brain death, recovery procedures and effects of anesthesia and surgery) that could have deleterious effects on renal function. One can speculate that the difference in the extent of damage within groups, if one exists, it is not related to blood flow changes, nor is it possible to assess using CEUS.

Although other reports [14, 28] have shown a significant difference in TIC parameters between good graft function and acute rejection, these indexes do not apply for differentiating DGF (without acute rejection) from EGF. Since our patients were not submitted to biopsy after transplantation, it was not possible to compare the results of CEUS according to the histological features. However, from a clinical point of view, the need for early dialysis is one of the more important issues following transplantation. Nonetheless, until now, CEUS has not been a standard examination in routine clinical practice to assess renal RBF yet in contrast to the increasing use to evaluate focal lesions.

A second important finding of this study is that early postoperative renal transplant recipients, in comparison to good functioning graft patients at least 90 days after surgery, showed a clear difference in TIC parameters. TTP and RT are parameters that mirror the wash-in of the contrast through the ROIs. Since TTP includes part of the contrast time traveling from the injection site to the ROI, it can be affected by prerenal factors, while RT reflects the enhancement process exclusively within the ROI [13]. However, in the current study, the two parameters showed a good correlation. Taken together, the differences in TTP and RT point towards a delayed time to reach the cortex peak and a shorter time to travel through the medulla in early group in comparison with the late group as a whole and especially with the subset of patients with creatinine levels in the lowest tertile (stable renal function). The longer time to reach the cortex peak is in accordance with the higher resistive index seen in the early group. On the other hand, a shorter time to reach the medullary peak and faster transit time through the medulla would be expected if a microcirculation shunt has taken place [30]. Vascular bypasses that give rise to a descending vasa reta [31], including continuous afferent-efferent vessels and short vascular connections between afferent and afferent arterioles [32], have been reported in approximately 10% of juxtamedullary glomeruli [31]. The role of possible arteriovenous fistulae following perioperative allograft biopsy has not been taken into consideration, but should also be kept in mind [33]. Within the late group, the comparison of TIC parameters between the lowest and highest creatinine tertiles provides further evidence of a change in regional RBF in renal dysfunction. Moreover, using continuous infusion of contrast agent, it has been shown that serum creatinine levels are inversely related to RBF in renal transplant recipients [11].

The weakness of this study is that no biopsies were taken after transplantation (besides peri-implantation wedge biopsies) to better differentiate the groups. Therefore, it is possible that the EGF and DGF subgroups were unequally contaminated by cases of acute rejection and/or acute tubular necrosis. However, it is well-known that using the current immunosuppressive regimen, the incidence of acute rejection in the first 2 weeks is as low as 2.28% (6/263) [34] and the median time to the acute rejection is 23 days [35]. Moreover, in the two groups, the kidney size and Doppler indices were fairly similar, providing additional evidence of the homogeneity of the two groups. Therefore, the likelihood of patient selection bias is negligible.

## Conclusion

In accordance with other reports, the results obtained in the current study point toward the ability of CEUS to detect changes in contrast enhancement in different kidney territories of the graft using TIC. Although clear differences were found in TIC parameters between early and late transplant patients, no blood flow differences between DGF and EGF patients could be demonstrated using CEUS in the early post-transplant period. These results seem to support that some approaches to increase renal blood flow in DGF are useless.

## Abbreviations

ATN: acute tubular necrosis
CEUS: contrast enhanced ultrasonography
CIT: cold ischemia time
DGF: delayed graft function
EGF: early graft function
HLA: human leukocyte antigen
IRI: ischemia-reperfusion injury
MI: mechanical index
RBF: renal blood flow
RI: resistive index
ROI: regions of interest
RT: rising time
TIC: time-intensity curve
TTP: time to peak
